# Geological and geophysical data compilation for the western Wabigoon and southern Abitibi subprovinces of the Superior Province, Ontario, Canada

**DOI:** 10.1016/j.dib.2021.107159

**Published:** 2021-05-21

**Authors:** Rebecca M. Montsion, Stéphane Perrouty, Ben M. Frieman

**Affiliations:** a Mineral Exploration Research Centre, Harquail School of Earth Sciences, Laurentian University, Sudbury, Ontario P3E 2C6, Canada; b Centre for Exploration Targeting, School of Earth Sciences, The University of Western Australia, 35 Stirling Highway, Crawley, 6009, Australia

**Keywords:** Field geological data, Wabigoon, Abitibi, Dryden, Timmins, Precambrian, Archean, Greenstone

## Abstract

The geoscientific data presented in this paper are a foundation for experimental and exploration geological research in the western Wabigoon and southern Abitibi subprovinces of the Superior Province in Ontario, Canada. New geological interpretations, in map and GIS formats, along with compiled mineral deposit information, structural databases, magnetic susceptibility measurements, and reprocessed aeromagnetic grids have been integrated to provide a basis for comparative studies between the two geologically similar yet economically disparate greenstone belts near Dryden and Timmins, Ontario, Canada. Data were acquired from a wide range of publicly sourced data releases and enhanced through the addition of new observations. New geological maps presented for both regions represent the culmination of integrating the multi-disciplinary geoscientific database and recent geological interpretation. Data contained within this publication are co-submitted with Montsion et al. [1].

## Specifications Table

**Table:** 

Subject	Geology
Specific subject area	Regional mineral exploration dataset
Type of data	[List the type(s) of data this article describes. Simply delete from this list as appropriate:] Table Excel spreadsheet ESRI shapefile (.shp) ESRI map project (.mdx) Geosoft grid (.grd) geoTiff (.tif) Map (.pdf)
How data were acquired	• Observational geological data (e.g., structural points, outcrop lithology) were digitized from scanned maps, compiled from open source files, or observed during recent field studies (2018–2020). • Interpreted geological data (e.g., fault traces, lithology polygons) were interpreted in ArcMap by integrating all available datasets such as compiled field observations, scanned legacy maps, geophysical grids, satellite imagery, and Quaternary maps. Only new, reprocessed, or compiled datasets are included herein. • Mineral occurrences were compiled from open access sources and enhanced by extracting information from all available NI43–101 reports and calculated totals. • Geophysical grids were retrieved from open access data repository and reprocessed in Seequent's Oasis Montaj
Data format	Compiled Observed Measured Interpreted Calculated
Parameters for data collection	Data was compiled and integrated at a scale appropriate for a regional mineral exploration study. The best resolution available for each dataset was included and then filtered or interpreted for fit with the map scale
Description of data collection	N/A (see description in ‘How data were acquired’)
Data source location	Institution: Laurentian University City/Town/Region: Sudbury Country: Canada Latitude and longitude (and GPS coordinates, if possible) for collected samples/data: Dryden: 49.10, −92.06, 49.91, −93.02; Timmins: 48.30, 49.75, −80.73, −81.61 List of primary data sources are listed and described in ‘Data sources for constraint and compiled layers’
Data accessibility	Repository name: Mendeley Data (https://data.mendeley.com/datasets/rvnk26krjs/2) Instructions for accessing these data: Use link to access and download all components
Related research article	Authors’ names: Rebecca M Montsion, Stéphane Perrouty, Mark D Lindsay, Mark W Jessell, Ben M Frieman Title: Mapping structural complexity using geophysics: A new geostatistical approach applied to greenstone belts of the Superior Province, Canada Journal: Tectonophysics https://doi.org/10.1016/j.tecto.2021.228889

## Value of the Data

• This dataset represents a comprehensive and up-to-date regional, open-access, geoscientific compilation for the western Wabigoon (centred on Dryden, Ontario, Canada) and southern Abitibi (centred on Timmins, Ontario, Canada) subprovinces
• All of the included datasets are intended for use by geologists conducting geoscientific studies in both or either of the areas of interest
• Included data can be used as a foundation for mineral exploration, regional-scale investigations, and comparative studies

## 1 Data Description

This comprehensive, multi-disciplinary geoscientific database provides access to observational, measured, and interpreted data for two Archean greenstone belts centred on Dryden, Ontario Canada in the western Wabigoon subprovince and on Timmins, Ontario, Canada in the southern Abitibi subprovince. Layers (e.g. point, line, polygon) with a spatial component are listed within the ‘Geology’ folder as ESRI shapefiles. Grid files representing reprocessed geophysical data are provided as .tiff and .grd files. These have been spatially projected into the NAD 83 UTM coordinate system (zone 15 N for Dryden and 17 N for Timmins). For each layer, metadata descriptions relating to symbology, source (if applicable), and other relevant information are recorded in the associated attribute table. Detailed descriptions of complied layers can be found in their respective source publications.

### 1.1 Geological

Geological layers consist of georeferenced symbols and shapes representing geological observations (e.g., structural measurements, mineral occurrences, main lithology at outcrop) and interpretations (e.g., faults traces, map units). Digitized and compiled structural measurements (‘Structural measurements’) and mineral occurrence locations (‘Mineral deposit index’) are presented as Excel tables in separate folders in a Mendeley Data repository [2]. Interpreted layers such as lithology polygons (‘MapUnits’) and structural traces (‘Geolines’) are provided as ESRI shapefiles in the ‘Geology’ folder and are organized according to their spatial location. References for data that constrained interpretations or contributed to compilations are reported in a separate folder of the Mendeley Data repository (‘Data sources for constraint and compiled layers’; [2]).

### 1.2 New Geological Maps

**Fig. 1:**
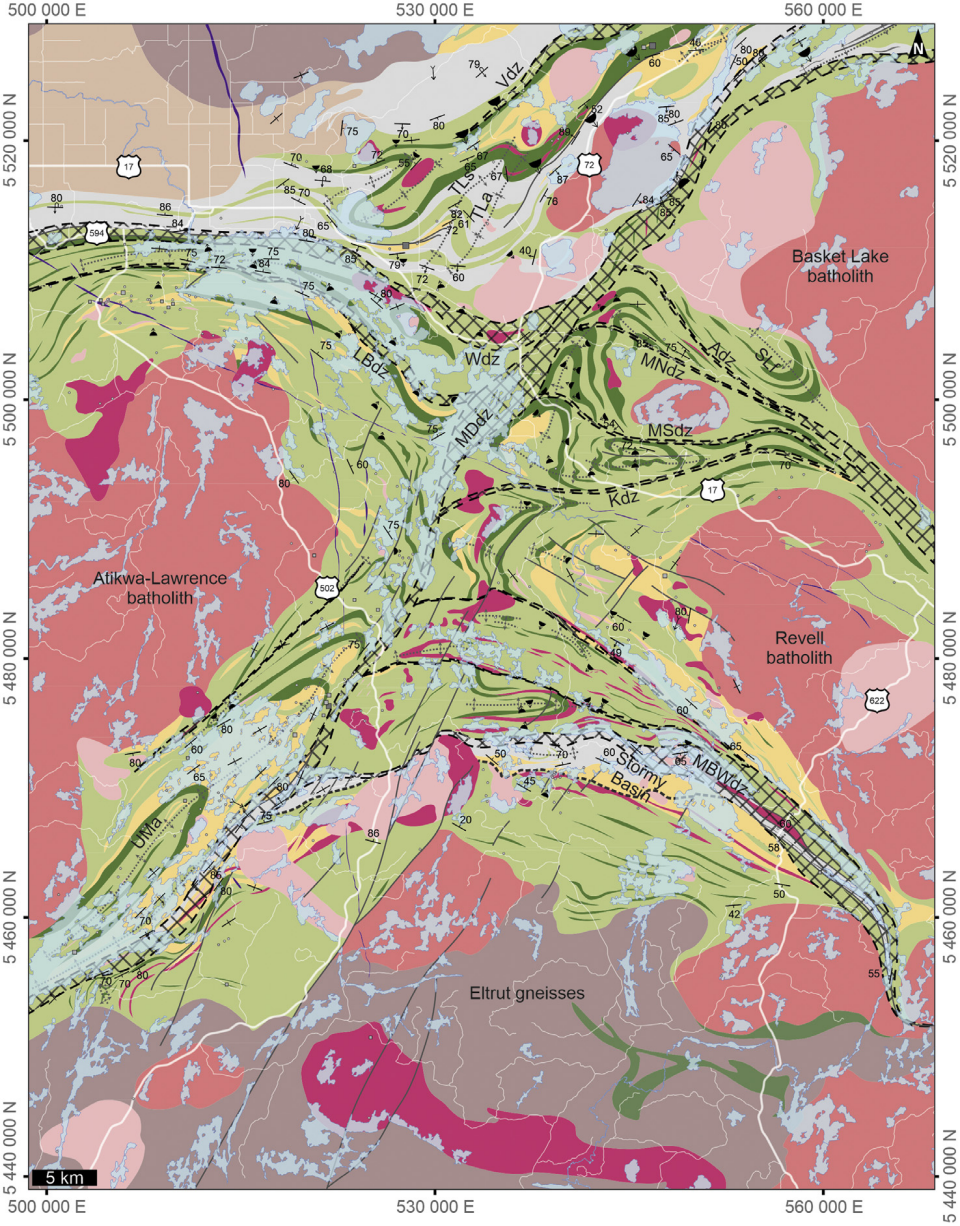

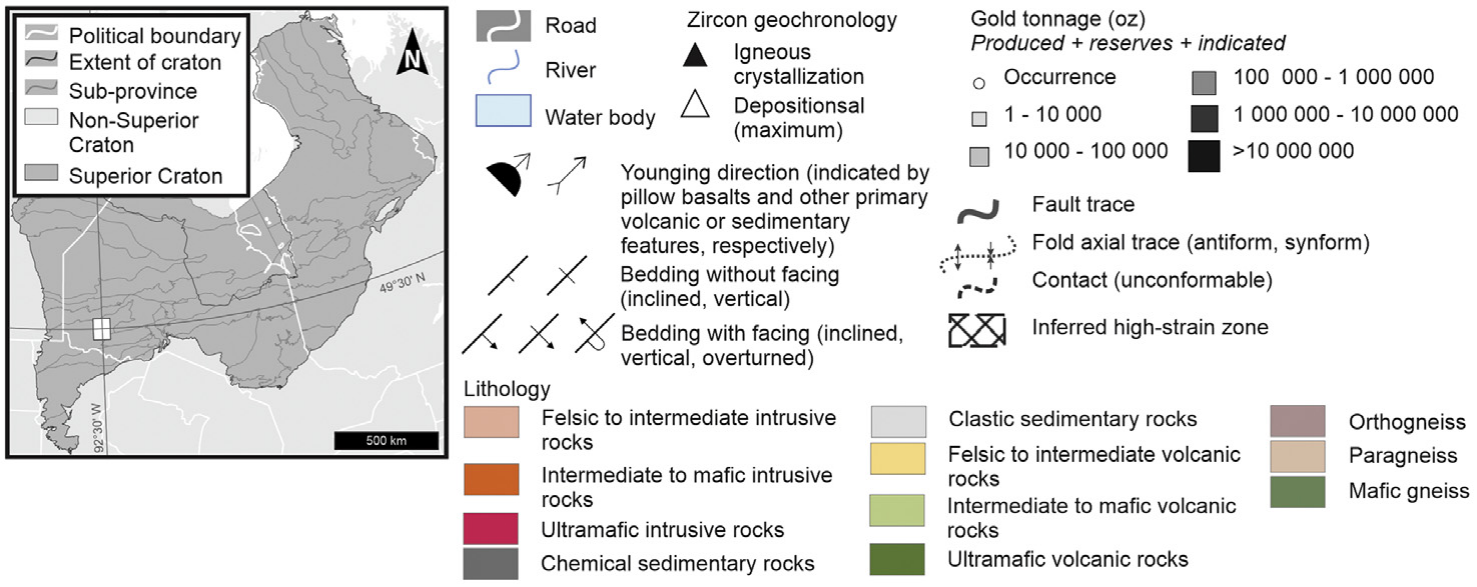
Geological map of the Dryden area, Ontario in the western Wabigoon subprovince of the Superior Province; Map displays polydeformed Archean bimodal volcanic stratigraphy with overlying sedimentary packages variably intruded by tonalitic to granitic plutons and smaller porphyritic bodies. Proterozoic diabase dikes crosscut stratigraphy as well as intrusive bodies; Geological interpretation compiled and integrated from 64 geological maps (listed in ‘Data sources for constraint and compiled layers’); Coordinates in NAD 83 UTM zone 15 N; Adz: Aiabewatik deformation zone; ALb: Atikwa-Lawrence batholith; BLb: Basket Lake batholith; Eg: Eltrut gneisses; Gld: Goldlund deposit; Gd: Goliath deposit; Kdz: Kawashegamuk deformation zone; Kd: Kenwest deposit; LBdz: Larson Bay deformation zone; LLRa: Long Lake River antiform; MDdz: Manitou-Dinorwic deformation zone; Mndz: Melgund north deformation zone; Msdz: Melgund south deformation zone; MBWdz: Mosher Bay-Washeibemaga deformation zone; NLdz: Noonan Lake deformation zone; Rb: Revell batholith; SB: Stormy Basin; SLa: Suzanne Lake antiform; TLa: Thunder Lake antiform; TLs: Thunder Lake synform; UMa: Upper Manitou antiform; VHd: Van Horne deposit; Vdz: Vermillion deformation zone; Wdz: Wabigoon deformation zone.

Two new geological maps, one for each map area, present a scale appropriate interpretation of geological data and relationships. Maps are available as PDF sheets (in ‘PDF Maps’) or as ESRI map project file (in ‘Geology’). The ESRI .mxd files are provided for comprehensive interrogation or to interact with individual layer files. The new geological map of the Dryden area, Ontario in the western Wabigoon subprovince of the Superior Province (Fig. 1) displays poly-deformed Archean bimodal volcanic stratigraphy with overlying sedimentary packages variably intruded by tonalitic to granitic plutons and smaller porphyritic bodies. Proterozoic diabase dikes crosscut stratigraphy as well as intrusive bodies. Geological interpretation integrated observations and interpretations from 64 geological maps (listed in ‘Data sources for constraint and compiled layers’), geophysical map patterns, and new field observations. Geochronological data from Meek et al. [3] were also used to constrain lithotectonic assemblage extents and assign map units. Map coordinates are in NAD 83 UTM zone 15 N./ Labelled features within the map include Aiabewatik deformation zone (Adz), Atikwa-Lawrence batholith (Alb), Basket Lake batholith (BLb), Eltrut gneisses (Eg), Goldlund deposit (Gld), Goliath deposit (Gd), Kawashegamuk deformation zone (Kdz), Kenwest deposit (Kd), Larson Bay deformation zone (LBdz), Long Lake River antiform (LLRa), Manitou-Dinorwic deformation zone (MDdz), Melgund north deformation zone (Mndz), Melgund south deformation zone (Msdz), Mosher Bay-Washeibemaga deformation zone (MBWdz), Noonan Lake deformation zone (MLdz), Revell batholith (Rb), Stormy Basin SB), Suzanne Lake antiform (Sla), Thunder Lake antiform (TLa), Thunder Lake synform (TLs), Upper Manitou antiform (UMa), Van Horne deposit (VHd), Vermillion deformation zone (Vdz), Wabigoon deformation zone (Wdz).

**Fig. 2:**
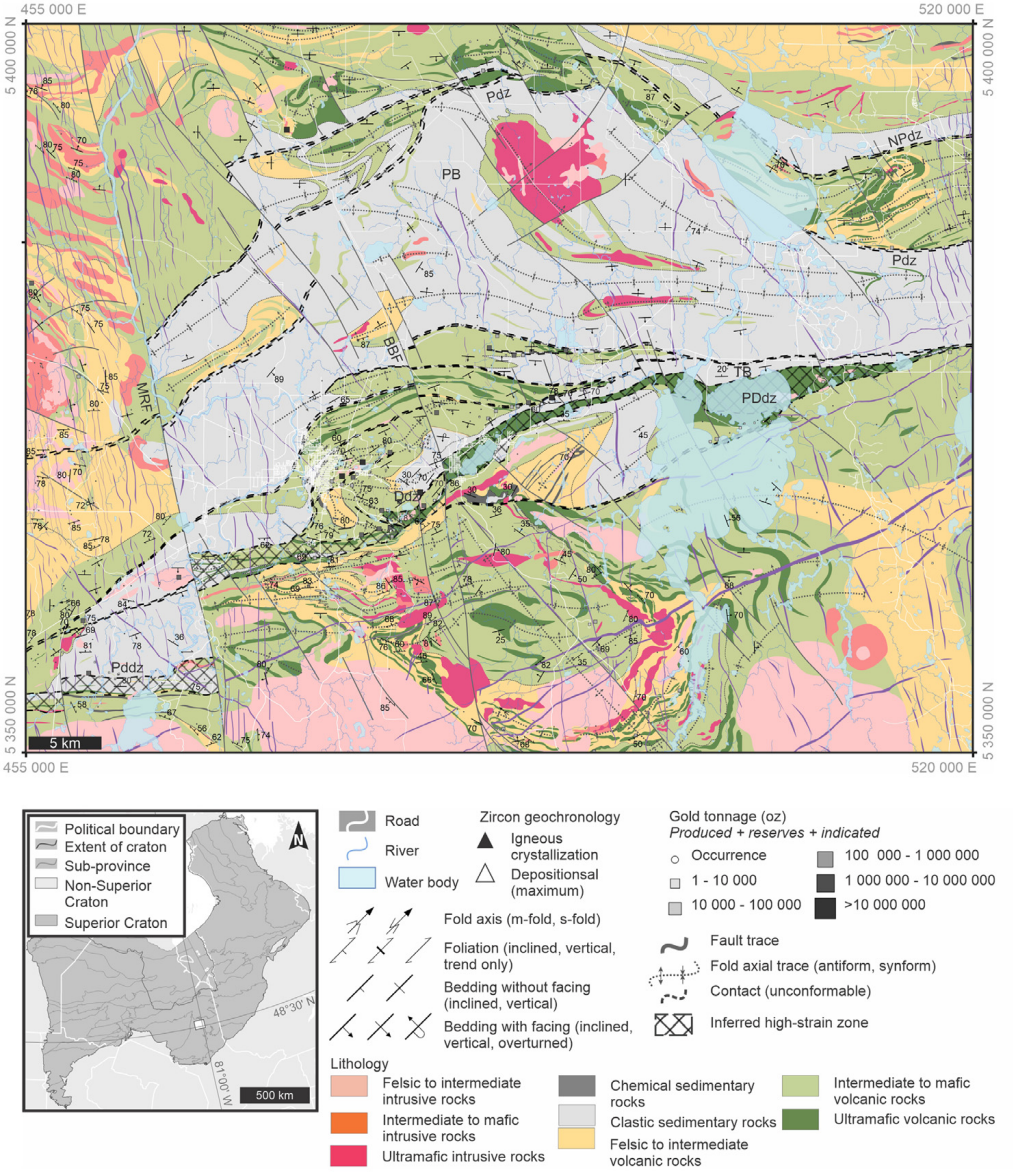
Regional geological map near Timmins, Ontario in the southern Abitibi subprovince of the Superior Province; Map displays poly-deformed Archean bimodal volcanic stratigraphy with overlying sedimentary packages variably intruded by tonalitic to granitic plutons and smaller porphyritic bodies. Proterozoic diabase dikes crosscut stratigraphy as well as intrusive bodies; Geological interpretation compiled and integrated from 33 geological maps and several compilation databases (listed in ‘Data sources for constraint and compiled layers’); BBF: Burrows Benedict fault; Ddz: Dome deformation zone; MRF: Mattagami River Fault; NPdz: North Pipestone deformation zone; PB: Porcupine Basin; PDdz: Porcupine-Destor deformation zone; Pdz: Pipestone deformation zone; TB: Timiskaming Basin.

The new regional geological map near Timmins, Ontario in the southern Abitibi subprovince of the Superior Province (Fig. 2) displays poly-deformed Archean bimodal volcanic stratigraphy with overlying sedimentary packages variably intruded by tonalitic to granitic plutons and smaller porphyritic bodies. Proterozoic diabase dikes crosscut stratigraphy as well as intrusive bodies. Geological interpretations were made by integrating compiled observations and interpretations from 33 geological maps and several compilation databases (listed in ‘Data sources for constraint and compiled layers’), geophysical map patterns, and new field observations. Geochronological data from Meek et al. [3] were also used to constrain lithotectonic assemblage extents and assign map units. Labelled features within the map include the Burrows Benedict fault (BBF), Dome deformation zone (Ddz), Mattagami River Fault (MRF), North Pipestone deformation zone (NPdz), Porcupine Basin (PB), Porcupine-Destor deformation zone (PDdz), Pipestone deformation zone (Pdz), Timiskaming Basin (TB).

### 1.3 Structural Observations

In Dryden, 13,612 previously published structural measurements (e.g., bedding, younging, lineations, and foliations) were digitized from 64 geological maps and combined with 1047 new observations from 2018/2019 fieldwork.

In Timmins, 8047 structural observations were compiled and integrated from several open access data sources and 33 scanned maps listed in ‘Data sources for constraint and compiled layers’. The source of each point is recorded in ‘Data sources for constraint and compiled layers’. All structural data are released using a dip direction/dip format.

### 1.4 Economic Geology

The location of mineral occurrences and deposits were extracted from the Ontario Geological Survey's Mineral Deposit Index (2020). The compiled table includes 254 locations in Dryden and 537 locations in Timmins (‘Mineral deposit index’). Deposit name, grade, and tonnage were extracted where available; however, there were several gaps in quantitative records. Gaps in tonnage records were filled by extracting information from each deposit's NI43–101 disclosure for mineral projects reports. Gaps in grade (g/t and oz/t) and totals were calculated in the included Excel spreadsheet. The MDI identification number can be used to retrieve additional information from the Ontario Geological Survey website (https://www.geologyontario.mndm.gov.on.ca/MDI_Description.html).

### 1.5 Geophysical

Two geophysical datasets are included in this publication: 1) petrophysical measurements from recent field work in the Dryden area and 2) an ensemble of reprocessed aeromagnetic grids. These can be assessed from the ‘Geophysics’ folder in the Mendeley Data repository [2].

### 1.6 Petrophysical Data

A new dataset of magnetic susceptibility measurements from 2018 to 2019 field work in the Dryden area (‘Magnetic susceptibility’) reports results of 4022 readings collected at 809 outcrops, using a Terraplus KT-10 magnetic susceptibility meter. At each outcrop, several readings of the same rock type were recorded to minimize bias in the collected data and provides a more complete analysis of the rock's magnetic susceptibility. Magnetic susceptibility measurements in the Timmins area can be accessed from the Ontario Geological Survey website (https://www.mndm.gov.on.ca/en/mines-and-minerals/applications/ogsearth/precambrian-bedrock-magnetic-susceptibility-geodatabase).

### 1.7 Reprocessed Aeromagnetic Grids

An ensemble of reprocessed aeromagnetic grids (40 m by 40 m cell size) from the Ontario Geological Survey's [4] Stormy Lake and (2003) Timmins geophysical survey datasets are included as projected geo .tiff and .grd files. The ensemble includes total magnetic intensity reduced to pole with cosine roll off filtering, first and second vertical derivatives, tilt derivative, and dynamic range compression. Hill-shaded relief grids are also included, and their azimuth and angle of incidence are indicated in their filename. The original total magnetic intensity and reduced to pole aeromagnetic grids can be accessed from the Ontario Geological Survey website [4], [5].

## 2 Experimental Design, Materials and Methods

### 2.1 Geological mapping

Observational geological layers (e.g., structural measurements and mineral occurrence information) were compiled from several open source geoscientific compilations, digitized from archived scanned maps, extracted from geological reports, and supplemented with new observations. A summary table of methods, purpose, and sources for each data set is provided (‘Data sources for constraint and compiled layers’).

**Table 1:** Codes and schema for map units in Dryden and Timmins ArcMap project. Units and assemblages matched by approximate age and relative relatioships.

Wabigoon				Timmins		
Label	Description	Assemblage		Assemblage	Description	Label
Dd	Proterozoic age		**Diabase dikes**		Proterozoic age	dd

Idf	Felsic to intermediate		**Syn-deformation intrusions**		S-Type granites/ porphry suite	Idf
Idm	Mafic to intermediate				Mafic to intermediate	Idm
					Ultramafic to mafic	Idu

Ivd	Intermediate to felsic		**Syn-volcanic intrusions**		Intermediate to felsic	Ivf
Ivm	Mafic to intermediate				Mafic to intermediate	Ivm
Ivu	Ultramafic to mafic				Ultramafic to mafic	Ivu

Stormy_TS	Clastic sedimentary	Stormy	**Volcano-sedimentary units**	Timiskiming	Clastic sedimentary	TS_ms
Stormy_bif	Banded iron formation				Banded iron formation	TS_bif
Stormy_vf	Felsic volcanic				Felsic volcanic	TS_vf
Stormy_vm	Mafic volcanic				Mafic volcanic	TS_vm
TZB_ms	Porcupine-type	Thunder Lake / Zealand/Brownridge		Porcupine	Porcupine-type	PC_ms
TZB_bif	Banded iron formation				Banded iron formation	PC_bif
TZB_vf	Felsic volcanic				Felsic volcanic	PC_vf
TZB_vm	Mafic volcanic (low mag; high mag)				Mafic volcanic	PC_vm
TZB_vmhi						
				Blake River	Clastic metasedimentary	Blake_cls
Boyer_vf	Felsic volcanic	Boyer Lake			Chemical metasedimentary	Blake_chs
Boyer_vm	Mafic volcanic (low mag; high mag)				Felsic volcanic	Blake_vf
Boyer_vmHi					Mafic volcanic	Blake_vm
				Tisdale	Clastic metasedimentary	Tisdale_cls
					Chemical metasedimentary	Tisdale_chs
KW_vf	Felsic volcanic	Kawashegamuk / Wapageisi			Felsic volcanic	Tisdale_vf
KW_vm	Mafic volcanic (low mag; high mag)				Mafic volcanic	Tisdale_vm
KW_vmHi					Ultramafic volcanic	Tisdale_vu
				Kidd-Munro / Stoughton-Roquemaure / Deloro / Pacaud	Clastic metasedimentary	Kidd_cls
					Chemical metasedimentary	Kidd_chs
Wab_vf	Felsic volcanic	Wabigoon			Felsic volcanic	Kidd_vf
Wab_vm	Mafic volcanic (low mag; high mag)				Mafic volcanic	Kidd_vm
Wab_vmHi					Ultramafic to mafic volcanic	Kidd_vu

Og	Orthogneiss		**Basement**			
Pg	Paragneiss					
Bmg	Mafic gneiss					

### 2.2 Structural interpretations

For both the Dryden and Timmins databases, geological interpretations (e.g. fault traces, deformation zones, contacts, map units) were generated through an iterative process where interpretations from previous works at various scales (see source map citations from structural observations) were compared to structural data, dominant outcrop lithologies, geophysical grids, geochronological analyses, and mineral occurrence observations. Interpretations were continuously refined by targeted fieldwork and repeated comparisons to observational data. This iterative process is common for regional-scale geological investigations (Gunn et al., 1997; Aitken and Betts, 2009; Metelka et al., 2011; Perrouty et al., 2012; Isles and Rankin, 2013; Blundell et al., 2019).

During comparisons to geophysical grids, spatially continuous, roughly linear anomalies in aeromagnetic grids were assumed to represent variably magnetic volcanic (or sedimentary) layering. This assumption is supported by similarly oriented bedding measurements.

### 2.3 Map unit polygons

Map units were generated as polygons that outline the extent of a lithology type for a given unit within a litho-tectonic assemblage. Similar to structural interpretations, polygons were interpreted by iteratively cross-referencing all available observational data, geophysical interpretations, and previous works in the (i.e., scanned maps). Map units were classified based on lithology type and stratigraphy group using the schema in Table 1.

### 2.4 New geological maps

Precambrian bedrock and assemblage maps for both areas were generated by overlying observations and interpretations (filtered to be representative of geology at a 1:75 000 map scale).

### 2.5 Geophysical data processing

Raw data was acquired from the Ontario Geological survey and reprocessed as new geophysical grids for inclusion in this comprehensive database. For Dryden, aeromagnetic data were acquired between November 2000 and February 2001, with 200 m line spacing, 1500 m tie lines spacing, and a flight elevation of 70 m [4]. Timmins aeromagnetic data was acquired between 1975 and 1992, with 200 m line spacing, 1000 m tie lines spacing, and a flight elevation of 70 m [5]. Total magnetic intensity (TMI) data were gridded at 40 m resolution in both areas, using the minimum curvature algorithm in Seequent's Oasis Montaj™. Reduction to the pole (RTP) was based on the IGRF (International Geomagnetic Reference Field), calculated at the date of the survey (Dryden: declination −0.9°, inclination 75.2°; Timmins: declination −11.2°, inclination 74.8°). First and second vertical derivatives, tilt (Miller and Singh 1994; [6], [7]), and Phase Preserving Dynamic Range Compression [8], [9] were calculated from the RTP. Each technique increases contrast between anomalies and/or act as bandpass filters.

## CRediT Author Statement

**Rebecca Montsion:** Conceptualization, Methodology, Validation, Formal analysis, Investigation, Data curation, Writing - Original draft, Visualization; **Stéphan Perrouty:** Conceptualization, Methodology, Validation, Data curation, Writing - Review & Editing, Visualization, Supervision, Funding acquisition; **Ben Frieman:** Conceptualization, Validation, Data curation, Writing - Review & Editing, Visualization.

## Declaration of Competing Interest

The authors declare that they have no known competing financial interests or personal relationships which have, or could be perceived to have, influenced the work reported in this article.
